# Sequence divergence and diversity suggests ongoing functional diversification of vertebrate NAD metabolism

**DOI:** 10.1016/j.dnarep.2014.07.005

**Published:** 2014-11

**Authors:** Toni I. Gossmann, Mathias Ziegler

**Affiliations:** a Department of Animal and Plant Sciences, University of Sheffield, Alfred Denny Building, S10 2TN Sheffield, United Kingdom; b Department of Molecular Biology, University of Bergen, Postbox 7803, 5020 Bergen, Norway

**Keywords:** NAD metabolism, PARP, Positive selection, Pathway evolution

## Abstract

NAD is not only an important cofactor in redox reactions but has also received attention in recent years because of its physiological importance in metabolic regulation, DNA repair and signaling. In contrast to the redox reactions, these regulatory processes involve degradation of NAD and therefore necessitate a constant replenishment of its cellular pool. NAD biosynthetic enzymes are common to almost all species in all clades, but the number of NAD degrading enzymes varies substantially across taxa. In particular, vertebrates, including humans, have a manifold of NAD degrading enzymes which require a high turnover of NAD. As there is currently a lack of a systematic study of how natural selection has shaped enzymes involved in NAD metabolism we conducted a comprehensive evolutionary analysis based on intraspecific variation and interspecific divergence. We compare NAD biosynthetic and degrading enzymes in four eukaryotic model species and subsequently focus on human NAD metabolic enzymes and their orthologs in other vertebrates. We find that the majority of enzymes involved in NAD metabolism are subject to varying levels of purifying selection. While NAD biosynthetic enzymes appear to experience a rather high level of evolutionary constraint, there is evidence for positive selection among enzymes mediating NAD-dependent signaling. This is particularly evident for members of the PARP family, a diverse protein family involved in DNA damage repair and programmed cell death. Based on haplotype information and substitution rate analysis we pinpoint sites that are potential targets of positive selection. We also link our findings to a three dimensional structure, which suggests that positive selection occurs in domains responsible for DNA binding and polymerization rather than the NAD catalytic domain. Taken together, our results indicate that vertebrate NAD metabolism is still undergoing functional diversification.

## Highlights

• This study shows the role of natural selection in eukaryotic NAD metabolism.
• There is substantial heterogeneity in the evolutionary rates.
• NAD biosynthetic enzymes show stronger evolutionary constraint.
• NAD degrading/signaling enzymes evolve more rapidly and undergo positive selection.
• PARP family members show signatures of an ongoing functional diversification.

## 1 Introduction

Understanding how selection and genetic drift have shaped the composition of proteins is a central problem in population genetics and molecular evolution. Considering the complex interplay of enzymatic proteins and the critical homeostatic equilibrium they have to maintain it is not surprising that most amino acid changing mutations affecting enzymes will be deleterious [1], [2]. Due to recent advances in DNA sequencing technology it is now possible to obtain whole genome information from numerous non-model organisms and mutational variation of multiple individuals of the same species at reasonable costs [3], [4]. Patterns of sequence divergence between species and variation in natural populations can be used to reveal underlying selective fitness effects of mutations and can help to understand how natural selection has shaped the functions of enzymes and other proteins [2], [5], [6].

Enzymes are often involved in a complex network of interactions [7]. Various theories exist which aim to explain how complex enzyme networks can evolve [8]. A currently favored model, the patchwork model [9], [10], proposes that pathways evolve from a system of enzymes with a broad substrate specificity to enzymes with specialized functions through gene duplications. The rate at which a particular enzyme is evolving within a network depends on a number of factors, including how its products are involved in interactions with other gene products and where these products are located within the network [11], [12]. It has been proposed that the position of an enzymes within a metabolic pathway (e.g. upstream or at a branch point) may play a role, even though the models are restricted to relatively simple pathways [13], [14]. In brief, evolutionary theory suggests that important enzymes tend to accumulate fewer deleterious substitutions and undergo positive selection more frequently [13].

**Fig. 1:**
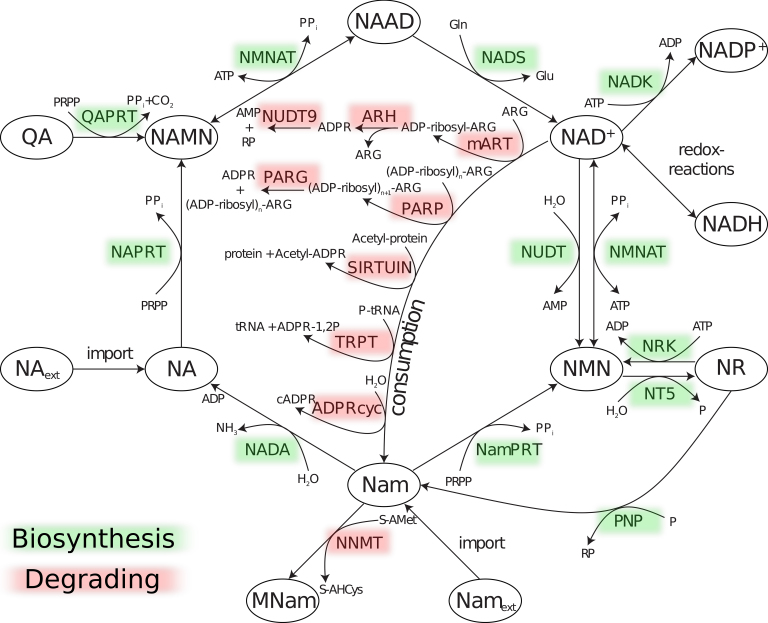
Schematic representation of the NAD pathway model. Enzymes are subdivided into biosynthetic (green) and degrading enzymes (red). NAD key metabolites are circled, remaining substrates and products are indicated by reaction arrows. For further details and abbreviations see [16]. (For interpretation of the references to color in this figure legend, the reader is referred to the web version of this article.)

NAD (nicotinamide adenine dinucleotide) is a key metabolite [15] as it participates in a vast number of redox reactions, but is also involved in a variety of signaling reactions. In contrast to redox reactions, these signaling reactions include degradation of NAD and require a permanent replenishment of cellular NAD pools. In yeast and human, NAD can be synthesized via a number of possible routes [15] which illustrates the fact that NAD metabolism can be considered as a relatively complex metabolic network (Fig. 1). From a phylogenetic perspective the occurrence of NAD biosynthetic enzymes is remarkably conserved across taxa [16], with only a few exceptions [17], [18]. However the diversification of vertebrates species is associated with an diversification of NAD degrading enzymes [16]. In particular, mammals, including humans, have numerous NAD degrading enzymes. Because of the physiological importance of NAD signaling, the effect of the constant turnover of NAD on NAD biosynthesis has gained increased attention over the past years [19], [20], [21].

ADP-ribosyltransferases constitute the largest group of NAD degrading enzymes. They transfer the ADP-ribose moiety of NAD onto acceptor proteins coincident with the release of nicotinamide [22]. ADP-ribosyltranferases can be distinguished as either mono-ADP-ribosyltransferases (mARTs) or poly-ADP-ribosyltransferases (PARPs), even though this classification is inaccurate [22], since it is unclear if all PARPs truly involve a poly-ADP-ribose catalytic function. However, different PARPs have been experimentally proven to be involved in enzymatic processes such as DNA repair, maintenance of genomic stability and response to oxidative stress [23]. The PARP family consists of 17 family members, but the majority of poly-ADP-ribose is synthesized by PARP1 [24] and some PARP family members remain poorly characterized experimentally [22].

Even though the metabolic and biochemical properties of human NAD metabolic enzymes have been intensely studied, there is currently a lack of a systematic evolutionary analysis of how natural selection has shaped sequence patterns of NAD metabolic enzymes. Because of the complexity of the NAD metabolic network (Fig. 1) it is difficult to make clear predictions on how evolutionary forces have shaped the sequence composition of NAD metabolic enzymes. Given the universality of NAD, its biosynthesis and the physiological importance of NAD degrading enzymes it seems plausible that mutations affecting NAD metabolic enzymes are likely to introduce harmful changes leading to enzymatic malfunction. However based on the distribution of NAD metabolic enzymes across taxa [16] it becomes apparent that vertebrates harbour NAD degrading enzymes that are absent in other species. These “new” enzymes are likely to be the result of duplication events and therefore may gain new or modified functions according to the patchwork model [25], [26]. From an evolutionary perspective, phylogenetically young genes tend to evolve under relaxed selective constraint and possibly undergo adaptation more frequently [27]. We therefore postulate that among the genes encoding NAD degrading enzymes there are candidates that should show signatures of positive selection or the random accumulation of mutations as a part of subfunctionalization and therefore show a higher rate of protein evolution.

In this study we test the hypothesis that NAD biosynthetic and degrading enzymes show differences in their evolutionary patterns. We start our analysis on four model species, humans, mice, *Drosophila* and *Arabidopsis* and present evidence for differences in divergence and diversity between the two enzyme groups. We show that the direction of selective constraint is inconsistent between the species, which may reflect differences in their effective population sizes. Based on 50 human NAD metabolic enzymes, we subsequently calculate evolutionary rates and determine the role of positive selection for vertebrate enzymes involved in NAD metabolism. We particularly focus on two members of the PARP family, PARP4 and PARP14. Human PARP4 is an enzyme with an unusually large number of high frequency segregating variation and shows a strong signature of population differentiation, as well as a possible signature of population specific positive selection. We reveal that the signature of population differentiation and positive selection coincides with the end of a homologous region which undergoes unequal recombination with an adjacent pseudogene. Secondly we provide strong evidence that PARP14 has been undergoing recurrent events of positive selection within the vertebrate clade. We identify potentially positively selected sites that lie in the functional domains of the PARP14 protein, but outside its catalytical domain, and are likely to have an impact on regulation and target substrate binding. We conclude that among NAD degrading enzymes there are fast evolving enzymes, illustrating an ongoing functional diversification in mammalian NAD metabolism.

## 2 Materials and methods

### 2.1 NAD pathway model

We focused on 50 human NAD metabolic enzymes based on a previous study and the availability of sequence data [16], a detailed list of gene identifiers can be found in Supporting dataset S1. We did not include human PARG in the study as we could not identify the gene in the HapMap dataset. We classify enzymes as either NAD biosynthetic or NAD degrading (Fig. 1) depending on whether they are part of a biosynthetic pathway or not [15].

### 2.2 Model species comparisons

We obtained sequence divergence and diversity of four model species: humans, *Arabidopsis thaliana*, *Drosophila melanogaster* and mice [16]. Sequence information was retrieved as follows: For humans we obtained information of the human genome project [28], coding SNPs for each gene were extracted from > 1000 human individuals and were resampled to 20 individuals using binomial sampling which is a reasonable approximation due to the large sample size. We also use this information to identify SNPs that segregate at high frequency (> 20%) within the human population. Divergence was obtained using the genome of macaque [29] to avoid misinference by segregating polymorphisms [30]. For *Drosophila* we used sequencing information from 17 sub-Saharan individuals [31] and outgroup information to *D. yakuba* [32], for *Arabidopsis* we used sequence information from 80 *Arabidopsis thaliana* accessions [33] and outgroup information to *A. lyrata* [34] and sequence information from ten wild house mice (*Mus musculus castaneus*) individuals [35] using rat as outgroup [36]. We focus on protein coding parts and obtain the number of nonsynonymous polymorphisms (*P*_*n*_) and synonymous polymorphims (*P*_*s*_) as well as the number of nonsynoymous substitutions (*D*_*n*_) and synonymous substitution (*D*_*s*_) for each gene. Because some genes are absent in a species or have little or no polymorphism or substitutions we sum data across genes for the two enzyme groups, NAD biosynthetic or NAD degrading. Estimates for *D*_*n*_ and *D*_*s*_ were calculated using codeml package of PAML (runmode = −2) or in case of mouse-rat comparison provided from the authors [35] as the number of fourfold and zerofold divergent sites. Significance for the contrast of *P*_*n*_/*P*_*s*_ and *D*_*n*_/*D*_*s*_ between enzyme groups was addressed by permutation tests. A classic McDonald Kreitman (MK) test [5] was conducted by contrasting patterns of polymorphisms and divergence. Within the MK framework an excess of nonsynonymous divergence to nonsynonymous polymorphisms suggests the action of adaptive evolution.

### 2.3 Rates of molecular evolution of vertebrate NAD metabolic enzymes

We identified orthologous sequences for the 50 human enzymes using whole genome information from 22 vertebrate species which we obtained from Ensembl [37] (full species list in Supporting dataset S1). To identify homologs we used the best BLAST hit [38] followed by a filtering procedure. We excluded sequences which were too long or too short (>20% in comparison to the human sequence) and for which too many positions did not have sequence information (>5 nt missing). Therefore the number of outgroup sequences varies for the 50 enzymes, either because a particular gene is absent or poorly sequenced. The remaining sequences were aligned with MAFFT [39] and unreliable alignment positions were removed with GUIDANCE [40]. The final alignment was processed with PAL2NAL [41] and used as input for PAML [42]. Synonymous mutations can be regarded as neutral or nearly neutral [43], and this characteristic may be used to infer selective effects of amino acid altering mutations [5], [44], [45], [46], [47]. The rate of molecular protein evolution can be measured by the ratio *ω* = *d*_*n*_/*d*_*s*_, where *d*_*n*_ and *d*_*s*_ are, respectively, the numbers of nonsynonymous and synonymous differences per nonsynonymous and synonymous site between two sequences [48], [49], [50]. PAML uses a maximum likelihood approach to obtain *ω* estimates for the provided phylogeny using certain model assumptions. To obtain *ω* for each set of orthologous enzymes we assumed a constant *ω* for the whole phylogeny (model M0, one-ratio model). We also conducted site tests which assumes heterogeneity in *ω* within genes. For this a likelihood ratio test (LRT) is conducted to address a significant fit of a more complex model in comparison to a nested simpler model. Evidence of positive selection is based on the comparison of the site model models M1a and M2a [51], [47] by applying an LRT with degrees of freedom (d.f.) = 2 and *P*< 0.001 which corresponds to a P-value of *P* = 0.05 with Bonferroni correction for multiple testing. Models M1a and M2a assume two classes of sites, either purifying selection (*ω*< 1) or neutrality (*ω* = 1), but M2a additionally allows for sites with *ω*> 1. To identify specific sites with *ω*> 1 we used the Naive Emprical Bayes (NEB) procedure as implemented in PAML and selected sites with a posterior probability > 0.5 to have *ω*> 1.

### 2.4 PARP4: *Fst* and *H*-statistic

To investigate patterns of polymorphism of the human PARP4 gene we included noncoding regions (e.g. UTR and introns). We calculated Fst [52], a measurement of population differentiation, using the R-package Popgenome [53]. Additionally, we calculated Fay and Wu's *H* statistic [54] for each of the human population separately using the software package DHtest (http://zeng-lab.group.shef.ac.uk). Fay and Wu's *H* measures an excess of high frequency derived variants in comparison to intermediate alleles, which should be present if recombination occurs during a selective sweep [55]. We obtained genomic information covering all exons and introns of human PARP4 on chromosome 13 spanning a region of 85 kb for 1092 individuals from 14 human population using the Hapmap data [28]. Test statistics (Fst and Wu's *H*) were calculated for overlapping sliding windows (3 Kb window size and step size 0.5 Kb). Confidence intervals for Fst values were obtained by calculating minimum and maximum Fst of 100 random subsamples of 546 individuals. PARP4 is also undergoing gene conversion from a nearby pseudogene (Dumont et al, personal communication), e.g. as a result of ectopic recombination [56]. The PARP4 pseudogene is located around 5 Mb before the PARP4 gene on chromosome 13 covering a region of 32 Kb and shares 28 SNPs with PARP4, of which two are coding (Supporting dataset S1)

## 3 Results

Here we conducted a comprehensive evolutionary analysis of NAD metabolic enzymes by using intraspecific variation and sequence divergence to other species. We restricted our analyses to the most common type of mutations, point mutations. In particular, we tested the hypothesis that NAD degrading enzymes show faster rates of protein evolution in comparison to NAD biosynthetic enzymes. First, we compared the evolutionary patterns of NAD biosynthetic and degrading enzymes in four model species. We used a general metabolic model derived from humans and yeast [15] and classified enzymes as either being NAD biosynthetic or NAD degrading (Fig. 1). We subsequently focused on human NAD metabolism, based on sequence variation and divergence of 50 human NAD metabolic enzymes to elucidate candidate enzymes with sequence patterns that are consistent with an evolutionary functional transition.

### 3.1 Evolutionary patterns of NAD metabolic enzymes vary across model species

As an initial analysis we compared intra- and interspecific protein coding sequence variation of NAD metabolic enzymes in four model species (human, mouse, *Drosophila* and *Arabidopsis*) for which data is publicly available. A measurement of the currently acting selective forces can be obtained by contrasting the ratio of nonsynonymous polymorphism to synonymous polymorphisms (*P*_*n*_/*P*_*s*_). Assuming that nonsynonymous mutations are largely deleterious or neutral, a relaxed action of purifying selection or an increased number of effectively neutral mutations would lead to higher *P*_*n*_/*P*_*s*_ values. Indeed, we found that in *Drosophila melanogaster P*_*n*_/*P*_*s*_ is significantly higher for enzymes which consume NAD than for biosynthetic enzymes (Table 1). The same trend is observed in the other three species, however not significant. One possible reason may be a lack of statistical power because the number of polymorphisms is relatively low and the number of protein sequences is limited by the design of the study.

The ratio of nonsynonymous substitutions to synonymous substitutions (*D*_*n*_/*D*_*s*_) is a measurement of the evolutionary rate of protein evolution. Relaxed purifying selection or frequent positive selection lead to an elevation of *D*_*n*_/*D*_*s*_, because nonsynonymous mutations tend to become fixed, i.e., replace the initial allele in the whole population, more frequently. Therefore it is important to disentangle the predominant type of selection, which however is not trivial [57]. Regarding NAD metabolic enzymes, we found that *D*_*n*_/*D*_*s*_ is significantly different in three species – humans, mice and *Arabidopsis* (Table 1). In humans and *Arabidopsis* there is an increased rate of *D*_*n*_/*D*_*s*_ for NAD degrading enzymes in comparison to NAD biosynthetic enzymes, but for mice the trend is the opposite. A possible explanation for this pattern are differences in the effective population size (*N*_*e*_, reviewed in [58]) between these species, because higher levels of *N*_*e*_ facilitate the action of selection. Humans and *Arabidopsis* have relatively small effective population sizes [30] and show little evidence for adaptive evolution [6], [59]. In contrast, *N*_*e*_ of wild mice is relatively large and as a consequence selection is more efficient [60]. Indeed, mice undergo frequent adaptive evolution [60], [35] which leads to increased levels of *D*_*n*_/*D*_*s*_. To test if positive selection has contributed to the divergence of NAD metabolic enzymes we contrasted patterns of polymorphisms and divergence (MK-test, [5]). We found evidence for positive selection in mice and *Drosophila* (Table 1), both species with large *N*_*e*_ and in the case of *Drosophila* this is only significant for NAD biosynthetic enzymes, but not NAD degrading enzymes. Theses results suggest that differences in *D*_*n*_/*D*_*s*_ in mice are largely caused by increased rates of adaptation, rather than relaxed levels of purifying selection. In contrast, for humans and *Arabidopsis* the differences between NAD degrading and NAD biosynthetic enzymes are likely caused by differences in the intensity of purifying selection. As a consequence, the role of positive selection in human NAD degrading enzymes appears limited, however it may be obscured by the dominating action of purifying selection.

**Table 1:** Diversity (*P*_*n*_/*P*_*s*_) and divergence (*D*_*n*_/*D*_*s*_) information for NAD biosynthetic and NAD degrading genes in four species. A test of positive selection (McDonald Kreitman (MK) test) was conducted, which contrasts the level of *P*_*n*_/*P*_*s*_ and *D*_*n*_/*D*_*s*_.

Species		NAD biosynthesis		NAD degradation
*Homo sapiens*				
	*P*_*n*_/*P*_*s*_	0.28	≈	0.45
	*D*_*n*_/*D*_*s*_	0.26	<	0.39
	MK-test	n.s.		n.s.

*Mus musculus*				
	*P*_*n*_/*P*_*s*_	0.10	≈	0.12
	*D*_*n*_/*D*_*s*_	0.32	>	0.26
	MK-test	**		**

*Drosophila melanogaster*				
	*P*_*n*_/*P*_*s*_	0.03	<	0.06
	*D*_*n*_/*D*_*s*_	0.06	≈	0.06
	MK-test	*		n.s.

*Arabidopsis thaliana*				
	*P*_*n*_/*P*_*s*_	0.21	≈	0.24
	*D*_*n*_/*D*_*s*_	0.12	<	0.16
	MK-test	n.s.		n.s.

n.s. – not significant; <, > signs indicate significant differences between the two enzyme groups using a permutation test; ≈ indicates no significant difference.

* *P* < 0.05.

** *P* < 0.001.

### 3.2 Analysis of substitution rate variation mammalian species

**Fig. 2:**
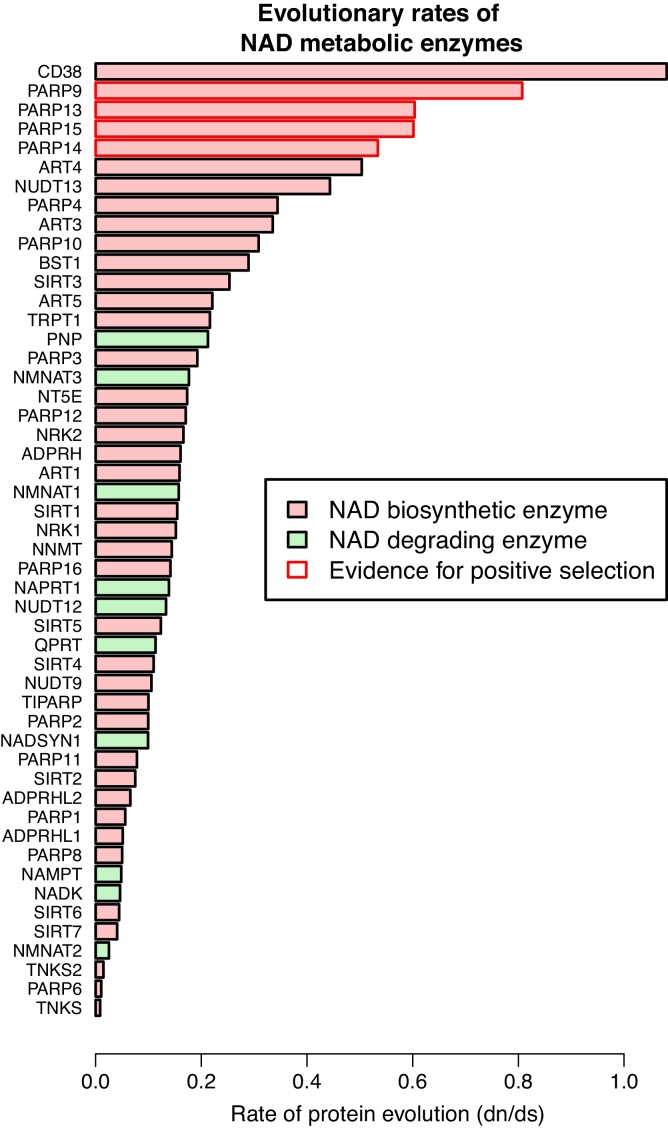
Evolutionary rates for 50 NAD metabolic enzymes measured as *d*_*n*_/*d*_*s*_ based on human NAD enzymes and their orthologs in other vertebrate species. Genes that show significant evidence for positive selection based on site test (see Section 2) are boxed in red. Parameter estimates for the site test results are shown in Table 2. (For interpretation of the references to color in this figure legend, the reader is referred to the web version of this article.)

**Fig. 3:**
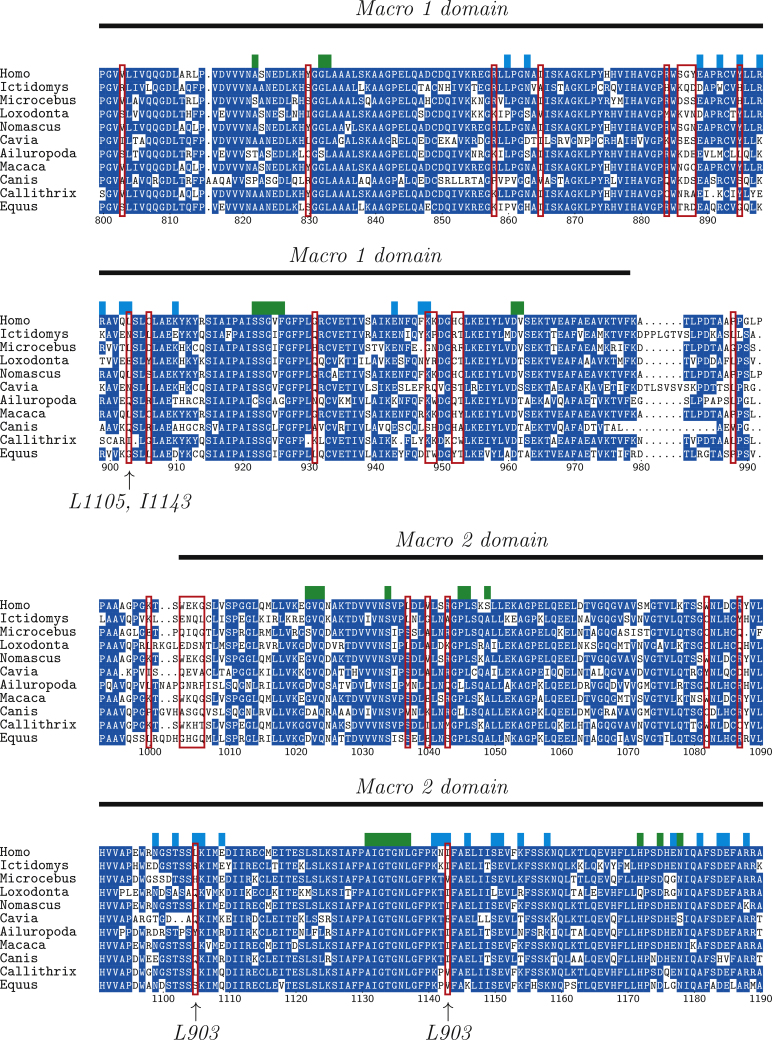
Alignment of PARP14 homologs covering macro domain 1 and macro domain 2 (human residues 800–1200). Red boxes indicate sites that have been identified to be evolving under positive selection. Green blocks indicate sites for substrate binding and cyan blocks indicate macro domain 1 and 2 interacting sites [62]. Site 903 has nonpolar interactions with sites 1105 and 1143. (For interpretation of the references to color in this figure legend, the reader is referred to the web version of this article.)

To measure the rate of evolution we use nonsynonymous to synonymous substitution rates (*ω*) which provides a sensitive measure of selective pressure at the protein level. *ω* values <1, = 1, and >1 indicate purifying selection, neutral evolution, and positive selection, respectively. We obtained orthologous sequences from vertebrate species for 50 human NAD metabolic enzymes (see Section 2) and calculated evolutionary rates assuming a constant *ω* between species (one-ratio model). *ω* ratios vary greatly between enzymes (Fig. 2) from *d*_*n*_/*d*_*s*_ = 0.008 for Tankyrase to *d*_*n*_/*d*_*s*_ = 1.08 for CD38. The top 14 enzymes with the highest *ω* ratios are NAD degrading enzymes. Regarding the NAD biosynthetic enzymes PNP and NMNAT3 show the highest evolutionary rates, NADK and NMNAT2 show the lowest rates. Since variation in *ω* ratio can be the result of differences in the type of selection, we investigated the role of positive selection. To identify whether increased rates of evolution are caused by events of positive selection, we conducted site-specific tests with PAML and found evidence for positive selection in four enzymes of which all belong to the NAD degrading enzymes (Fig. 2 and Table 2). Interestingly, all four enzymes are members of the PARP family (see 1), and in particular, PARP9, PARP14 and PARP15 form the group of macro domain containing PARPs. The macrodomain was initially described in histones and is involved in epigenetic regulation as a metabolite sensor on chromatin [61]. Recently a three dimensional structure of PARP14 has been published with focus on its macrodomains including binding sites and interactions between macro domains 1 and 2 [62]. We pinpoint 27 sites that show a signature of positive selection and lie in the macro domains 1 and 2 (Fig. 3), which is a significant enrichment compared to the rest of the protein (Fisher exact test, *P* = 0.016).

**Table 2:** Human NAD metabolic enzymes with evidence for positive selection based on substitution rate analysis using vertebrate orthologs. Likelihood ratio test *P*-values are derived from the comparison of model M1a (nearly neutral model) and M2a (neutral+positive selection) to test for positive selection assuming hetereogeous *ω* among sites. *p* denotes the proportion of sites with w>1, the number of orthologous sequences is denoted by # Seqs.

Enzyme	LRT *P*-value^a^	# Seqs	Parameter estimates
PARP9	0.0	7	*p* = 0.13, *ω* = 4.33
PARP13	3.13 × 10^−13^	3	*p* = 0.05, *ω* = 15.05
PARP14	0.0	11	*p* = 0.06, *ω* = 3.41
PARP15	3.46 × 10^−7^	5	*p* = 0.07, *ω* = 4.65

a Site models M1a vs M2a.

### 3.3 Amino acid altering variation segregating in human populations

**Fig. 4:**
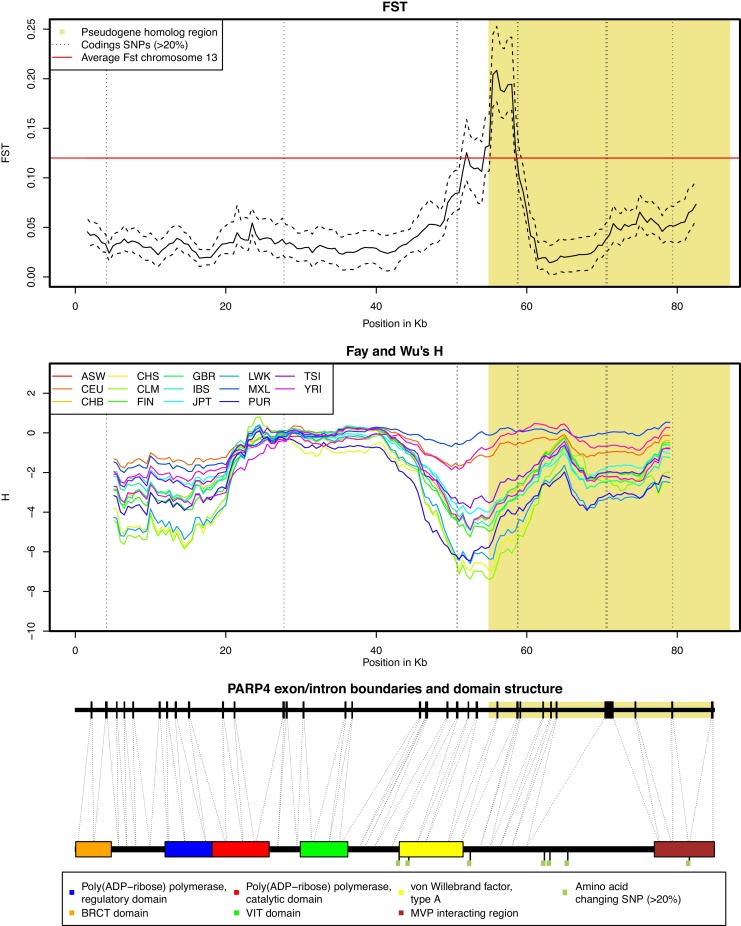
Sliding window analysis (3 Kb overlapping windows for FST and 10 Kb for H, step size 0.5 Kb) of human PARP4 variation. The upper panel shows FST, a measurement of population differentiation along with confidence intervals (dashed black line, obtained from extreme values of 100 subsamples) and average FST of human chromosome 13 [82]. The middle panel shows Fay and Wu's *H* statistic, a test statistic for positive selection in presence of frequent recombination. The lower panel indicates the PARP4 exon/intron distribution along with the proposed protein domains (InterPro Accession Q9UKK3 [80]) and MVP interacting region [64], [81]. (For interpretation of the references to color in this figure legend, the reader is referred to the web version of this article.)

We retrieved natural variation of human individuals from the Hapmap Genome project [28] to identify amino acid altering single nucleotide polymorphisms (SNPs) in NAD metabolic enzymes that are currently segregating in the human population at high frequencies (> 20%, Table 3). High frequency SNPs are likely to be polymorphisms which are either selectively neutral, under balancing selection or currently undergoing positive selection, but unlikely to have strong deleterious effects. Altogether we determined 21 high frequency amino-acid changing variants of which 4 belong to NAD biosynthetic enzymes (Table 3). The remaining 17 changes occur in ARTs and PARPs. Among the 21 high frequency mutations are 5 that are evolutionarily uncommon based on the PAM250 substitution score matrix [63] and they all occur in NAD degrading enzymes. Most strikingly we find that PARP4 (also dubbed vault-PARP [64]) has numerous high frequency nonsynonymous polymorphisms. A possible cause for this pattern is that PARP4 is currently undergoing a selective sweep [55] resulting in co-segregating SNPs. Another explanation could result from the fact that PARP4 has experienced gene conversion from a pseudogeneized paralog (Dumont et al., personal communication). Pseudogenes are genes that lost their protein coding ability and are therefore dysfunctional [65]. However, there are numerous polymorphisms at homologous positions that are segregating in both PARP4 and the PARP4 pseudogene likely caused by recombination events of the two different loci (ectopic recombination [56]). However, the region of non-homologous recombination does not entirely cover the PARP4 gene, but a stretch of around 38 Kb that includes the last 10 of the 33 PARP4 exons (e.g., the region encoding the C-terminus of the PARP4 protein, Fig. 4). To investigate these hypotheses in greater detail we retrieved haplotype information for the entire PARP4 gene along with the genomic alignment to chimpanzee. Based on a sliding window approach (see Section 2) we elucidated two remarkable patterns (Fig. 4). First there is a peak in the population structure, as measured by Fst, which is near the end of the nonhomologous recombining region. Second, we observe a signature of positive selection based on Fay and Wu's *H* statistic, which however appears to differ among the 14 populations. These population specific extreme values of *H* are also observed near the end of recombination region. Interestingly, we do not find an excess of diversity or divergence in the recombining region in comparison to the rest of the enzyme (results not shown).

**Table 3:** Amino acid changing mutations of human NAD metabolic enzymes currently segregating at high frequency (> 20%) in the human population. Evolutionary rare changes based on negative PAM250 scores are in bold.

Enzyme	Amino acid change (position)	Frequency
NRK	V ↔ M (358)	0.31
NADK	N ↔ K (262)	0.28
NT5E	T ↔ A (376)	0.76
PNP	G ↔ S (51)	0.22
ART1	**L** ↔ **P (257)**	0.55
ART3	**S** ↔ **L (341)**	0.47
ART4	D ↔ N (265)	0.28
PARP13	H ↔ Q (565)	0.26
	R ↔ K (485)	0.25
PARP15	**G** ↔ **R (628)**	0.23
PARP4	A ↔ P (1656)	0.42
	P ↔ T (1328)	0.38
	**G** ↔ **R (1280)**	0.38
	G ↔ A (1265)	0.38
	V ↔ A (1065)	0.64
	A ↔ T (899)	0.66
	S ↔ N (873)	0.23
PARP9	Y ↔ C (528)	0.54
PARP10	V ↔ A (630)	0.54
	**L** ↔ **P (395)**	0.35
	I ↔ V (249)	0.38

## 4 Discussion

Taking into consideration the centrality and universality of NAD and the fact that there is no known mechanism of extracellular uptake of NAD, it is clear that there is the need for cellular NAD synthesis during cell growth and a constant recycling of NAD to satisfy the needs of NAD dependent reactions. The importance of NAD metabolism is also reflected in evolutionary terms, because of the widespread abundance of NAD biosynthetic enzymes in almost all genomes that have been sequenced so far [66], [16]. However, within one organism multiple enzyme pathways may biosynthesize NAD, and the relative contribution of the different pathways to the overall cellular NAD content is still unknown [15]. Attempts to model pathways taking compartmentalization or even interactions between different tissues into account have been restricted to simple model systems as a consequence of lack of data due to experimental limitations [67], [68], [69]. Flux control analysis can predict the importance of an enzyme within a metabolic network [70]. However, at the current state there is clearly the need for a detailed analysis to link the outcome of flux distributions with an evolutionary interpretation [71].

A major predictor of an enzyme's evolutionary rate is its functional importance, but there are other factors which can influence patterns of sequence evolution of proteins, both within and between species [72]. This includes the local recombination rate, the linkage to nearby variation, epistatic effects and the effective population size (*N*_*e*_) [73], [30]. It is therefore difficult to derive clear predictions about how NAD metabolic enzymes are currently evolving and which evolutionary factors may have driven sequence divergence of NAD metabolic enzymes between vertebrate species. As a general assumption, we hypothesized that NAD biosynthetic enzymes should show a pattern of stronger selective constraint in contrast to NAD degrading enzymes which should reveal signatures of relaxed purifying selection and potentially show signs of functional diversification. In agreement with our prediction *P*_*n*_/*P*_*s*_, the number of nonsynonymous polymorphisms over synonymous polymorphisms, is consistently higher for NAD degrading enzymes in four model species. This illustrates that selection against deleterious mutations is less efficient or that more mutations tend to be effectively neutrally evolving in NAD degrading enzymes. The latter scenario is possible as a consequence of duplication events, under a model of subfunctionalization. Under such a scenario duplicated enzymes gain a specialized function and will therefore tend to evolve neutrally in the parts of the protein that do not exhibit a function anymore [26], [74]. NAD degrading enzymes, such as PARPs, SIRTUINs and ARTs form protein families that are likely to have originated from duplication events. We also observe significant differences in the evolutionary rates between the two enzyme classes in three of the four model species. However, they are inconsistent among species which is possibly a consequence of the differences in their *N*_*e*_. We showed that biosynthetic enzymes in humans and *Arabidopsis* exhibit reduced evolutionary rates, but appear to be evolving faster in mice, which may be attributed to the action of positive selection for which we find evidence in mice and *Drosophila*.

Based on substitution rate analysis of human metabolic enzymes along with their orthologs in other vertebrate species, we observe that the highest rates of protein evolution occur in NAD degrading enzymes, but it also becomes apparent that enzymes involved in biosynthesis are not consistently slowly evolving enzymes (Fig. 2). Even more surprisingly, there appears to be a substantial variation of evolutionary rate within both enzyme groups, NAD degrading and NAD biosynthesizing enzymes. One of the reasons for this heterogeneity may be caused by differences in the *N*_*e*_ of the different vertebrate species, as the number of species included differs for each of the enzymes due to technical (e.g. missing sequence information) or biological reasons (e.g. absence of an enzyme). Nevertheless, a cause for the observed heterogeneity in *d*_*n*_/*d*_*s*_ is the action of positive selection. We find evidence for four enzymes where positive selection has driven sequence divergence within the vertebrate lineage. Another aspect which explains the substantial variation is the unlikely assumption of a constant evolutionary rate within the whole phylogeny, as the rate variation may be influenced by few species. A large scale study of >16,000 human genes found around 2.4% of genes to be evolving under positive selection [75] in six mammalian genomes (human, chimp, macaque, mouse, rat and dog), which is significantly lower than 8% (binomial test, *P* = 0.03) identified in this study. This suggests two aspects, (1) NAD metabolism is enriched in genes undergoing positive selection and (2) positive selection may be in particular, acting in species that have not been included in the study of Kosiol et al. [75]. We find no evidence for positive selection based on branch specific test, as conducted previously [75], within the human lineage (results not shown).

Specific enzyme characteristics may cause evolutionary rate variation within proteins. There is striking evidence for evolutionary rate variation within vertebrate NMNATs [76], arguably the most central enzyme of NAD biosynthesis. The rate variation is caused by ISTIDs (isoform-specific targeting and interaction domains), which are interior stretches of the protein with an unknown three dimensional conformation. This potential conformational relaxation leads to elevated rates of protein evolution [76]. Indeed, NMNAT1 and NMNAT3 are among the fastest evolving NAD biosynthetic enzymes (Fig. 2), even though for two of the three ISTIDs a functional role has been experimentally verified [76]. However, protein families such as NMNATs and PARPs are not generally fast-evolving, e.g. NMNAT2 and PARP family members (Tankyrases and PARP6) are among the slowest evolving proteins. Among the slowest evolving enzymes is also NAMPT, the rate-limiting enzyme of NAD biosynthesis [77], [78], which was believed to be only present in vertebrates until very recently. However there is accumulating evidence, that this enzyme is present in lower eukaryotes and has been lost several independent times in various non-vertebrate lineages [16], [18]. However, enzymatic functionality of non-vertebrate NAMPT has yet to be shown.

Three macro-domain containing PARPs are among the enzymes with the highest evolutionary rates, and show a signature of positive selection. To identify potential functional roles of sites under positive selection we used a recently published three dimensional structure of PARP14 which includes binding sites as well as interaction sites of domains within the protein [62]. There are several sites subject to positive selection adjacent to binding sites, but more importantly, five macro domain interacting sites appear to have undergone positive selection (Fig. 3). Interestingly, three of the five sites form two nonpolar interactions between macro domain 1 and macro domain 2 [62], suggesting a potential coevolutionary mechanism. This suggest that not the NAD binding sites but the binding sites for the substrate on which the NAD moiety is transferred appears to be under evolutionary diversification. Taken together, the substitution rate analysis and the striking example of PARP14 shows a potential role of positive selection and illustrates that NAD degrading enzymes are under functional diversification. This also implies that experimentally proven functionality, e.g. in mouse, may not apply to other species, such as humans, and that a functional transfer from model species may be misleading.

Sequence variation in human NAD metabolic enzymes reveals that most amino acid changing SNPs are at very low frequencies suggesting strong deleterious effects of most of them. An exception is human PARP4 which shows an excess of high-frequency nonsynonymous polymorphisms. Based on a sliding window approach we observed a signal of population differentiation associated with a population specific negative value of Fay and Wu's *H* in PARP4, which may be interpreted as a signature of positive selection. Previous large scale analysis of human variation have not identified PARP4 as a target of selection [79] (http://haplotter.uchicago.edu/). One of the reasons may be that earlier approaches have been limited to the Hapmap2 dataset which was restricted to three human populations, while we use an extended dataset with 14 different populations. Interestingly there is evidence that PARP4 undergoes nonhomologous recombination with a nearby pseudogene (Dumont et al., personal communication) derived from common variation of PARP4 and the pseudogene. This pattern can, however, also be found in the chimpanzee genome and therefore may reflect a distant evolutionary event rather than ongoing nonhomologous recombination events. The signature of population differentiation coincides with the end of the nonhomologous recombination region, however it is difficult to address the biological significance. The PARP4 homologous region that shows shared variation of the PARP4 pseudogene covers the last 10 exons (≈700 aa), which is not assigned to any domain function according to the InterPro database [80] but the major vault protein (MVP) interacting region [64], [81].

## 5 Conclusion

Collectively, our results confirmed our general hypothesis that NAD biosynthetic enzymes are evolutionary more conserved while NAD degrading enzymes show less conservation and potentially are still undergoing functional diversification. However, we also showed that evolutionary patterns may differ between species and that there is a substantial evolutionary heterogeneity even within enzyme families (e.g. NMNATs, PARPs). We also stress that exceptional characteristics of particular enzymes may have an evolutionary impact, e.g. macro domains as targets for positive selection, the role of nonhomologous recombination or evolutionary heterogeneity within enzymes caused by ISTIDs. Taken together our findings provide a valuable example of how molecular evolution and population genetics can shed additional light on the functional characterization of enzymes and the networks they are involved in.

## Conflicts of interest

The authors declare that they have no conflicts of interest.
